# Achievement of another landmark during 2020 by Pakistan Journal of Medical Sciences

**DOI:** 10.12669/pjms.37.7.5208

**Published:** 2021

**Authors:** Shaukat Ali Jawaid, Masood Jawaid

**Affiliations:** 1 Shaukat Ali Jawaid Chief Editor, Pakistan Journal of Medical Sciences, Karachi - Pakistan; 2 Masood Jawaid Associate Editor, Pakistan Journal of Medical Sciences, Karachi - Pakistan

The Year 2020 has proved to be a great success for Pakistan Journal of Medical Sciences as we achieved yet another landmark of crossing the Impact Factor of one as per the Journal Citation Report released by Clarivate Analytics for the Year 2020. Our current Impact Factor is 1.088.^1^ In order to maintain the quality of the manuscripts accepted for publication we have also raised the bar gradually. Now routine research work has a very low priority with us. We are more interested in good quality research work with some innovations, manuscripts covering unique topics, something which adds new information to the world medical literature.

**Fig-1:**
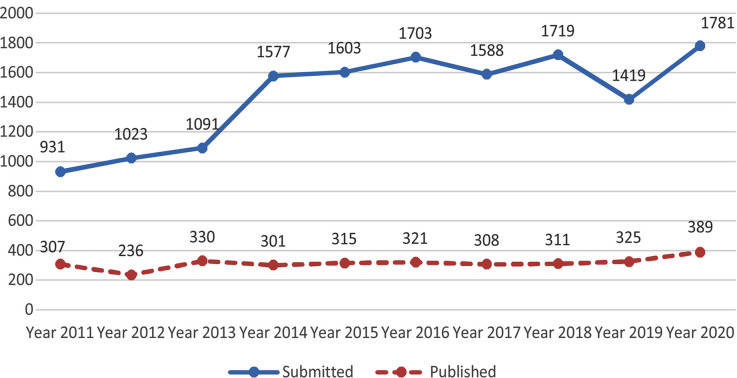
Submissions and Publication from Pakistan Pak J Med Sci (2011-2020).

A critical analysis of the year under review shows that the number of new submissions from Pakistan as well as overseas has also significantly increased. The total submissions in 2020 was 1781. While the number of submission are increasing but our acceptance rate has remained almost static about 20%. Fig-1. The number of manuscripts published during the same period were 389, thirteen papers were rejected after checking for plagiarism, twenty eight were withdrawn by the authors as they wanted urgent publication which we could not offer. Table-I and II.

**Table I:** PJMS manuscripts statistics of 2020 at a Glance.

Total Published Articles:	389
Articles not accepted for further processing	1217
Articles rejected due to Plagiarism:	13
Articles Withdrawn by Authors:	28
Under Process:	134
Total Received Articles:	1781

**Table II:** Country wise submissions during 2020.

*Country*	*Total*
Algeria	2
Australia	1
Bahrain	2
Canada	3
China	202
Colombia	2
Cyprus	3
Egypt	14
France	1
Georgia	1
Germany	1
India	10
Indonesia	29
Iran	40
Iraq	24
Ireland	3
Isle of Man	1
Italy	3
Jordan	9
Kazakhstan	3
Korea	7
Kuwait	2
Malaysia	9
Nigeria	11
Norway	3
Oman	1
**Pakistan**	**854**
Palestinian	1
Philippines	2
Portugal	1
Qatar	2
Russia	2
Saudi Arabia	144
Serbia	4
South Africa	2
Sudan	2
Thailand	3
Turkey	352
UAE	2
United Kingdom	12
United States of America	8
Viet Nam	1
Taiwan	1
Brazil	1

Grand Total	1781

**Fig-2:**
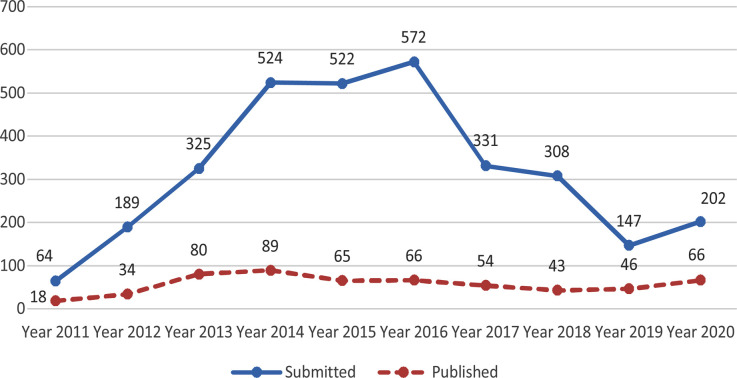
Submissions and Publication from China Pak J Med Sci (2011-2020)

The number of manuscripts received from Pakistan during 2020 were 854. Majority of them being from Karachi 261, Lahore 226, Islamabad 101, Peshawar 62 and Rawalpindi 43. Table-III. The number of manuscripts published during the Year-2020 increased to 389 as compared to 311 in 2018 and 325 in 2019. The number of submissions from China, Turkey and Saadia Arabia has been increasing which accounted for 202, 352 and 144 respectively. Table-II. A further analysis of submissions and publication of papers from these countries revealed that submissions from China were reduced from 2016 but it has now again started picking up as Chinese authors now take extra care while revising the papers responding to reviewers comments and suggestions which increases their chance of publication. Fig.2 On the contrary there has been drastic reduction in submission as well as publications from Islamic Republic of Iran over the years simply because the number of good quality standard medical journals published from Iran has increased.

**Table III:** City wise submissions from Pakistan during 2020.

*City*	*Total*
Abbottabad	2
Azad Kashmir	2
Bahawalnagar	1
Bahawalpur	12
Bannu	1
Chakdara	1
Charsadda	2
Dera Ghazi Khan	3
Dera Ismail Khan	2
Faisalabad	24
Gujranwala	2
Gujrat	6
Haripur	2
Hyderabad	19
Islamabad	101
Jamshoro	10
Karachi	261
Khairpur	3
Kharian	1
Lahore	226
Larkana	2
Mansehra	3
Mardan	4
Mirpur, Azad Kashmir	1
Mirpurkhas	1
Multan	26
Nawabshah	1
Nowshera	1
Pakpattan	1
Peshawar	62
Quetta	11
Rahim Yar Khan	1
Rawalakot	1
Rawalpindi	43
Sahiwal	1
Sargodha	3
Sialkot	4
Swat	3
Taxila	2
Wah Cantt.	2

Grand Total	854

**Fig-3:**
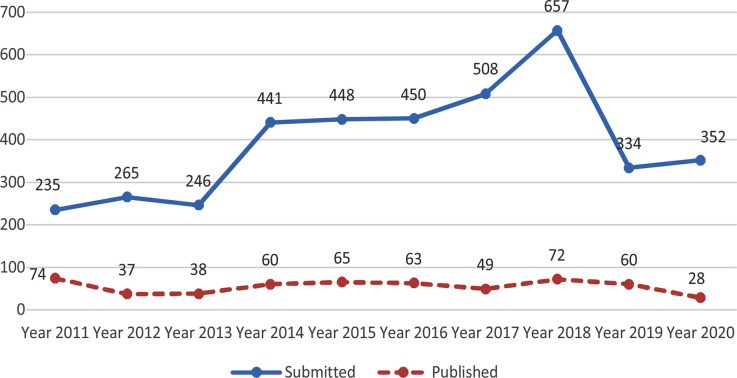
Submissions and Publication from Turkey Pak J Med Sci (2011-2020).

**Fig-4:**
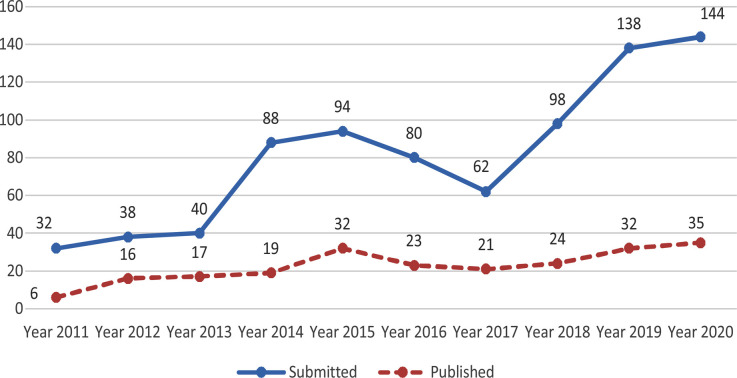
Submissions and Publication from Saudi Arabia Pak J Med Sci (2011-2020).

The number of submissions and publications from Turkey was increasing during the Years 2013-2018 but decreased in 2019 but has again increased in 2020. However, the number of papers accepted for publication after peer review has not increased much due to strict peer review Fig-3. Yet another country from where we attract large number of manuscripts for publication is Saudi Arabia. The number of submissions as well as papers accepted for publication has been increasing. Fig-4. However, more recently the authors from Saudi Arabia have been facing problems in transferring publication charges to Pakistan which has not only adversely affected the working of medical journals in Pakistan but may also deprive the country of precious foreign exchange.^2^ This is a serious issue which the Pakistan authorities needs to look into.

Researchers are under great pressure to publish their research work as soon as possible to meet deadlines for the completion of their research projects or to defend their PhD thesis but editors of biomedical journals in Pakistan are also faced with a dilemma due to human resource and financial constraints. Publications overseas in Impact Fact journals which the regulatory bodies in Pakistan as well as overseas demand is very expensive which many researchers cannot afford while the number of Impact Factor medical journals published from Pakistan so far remains just three. A few more journals have applied but it may still take some time and the authors who are always impatient will continue to suffer. The situation can be eased if the regulatory bodies in Pakistan like Higher Education Commission can accept Only Online Journals which will reduce the cost of production to a great extent and also enable the editors to accommodate more papers as there will be no page constraints. It will also reduce the publication charges for the authors which is expected to increase further in view of the devaluation of the Pakistani currency. 3

However, going for only Online Journals does involve some challenges as well in view of the increasing number of predatory journals. Hence, to check this menace, the authorities can give this permission of Only Online Journals to those publications which have earned Impact Factor, or indexed in Medline, and are covered by important databases like PubMed Central and Scopus. The regulatory bodies should also facilitate the journals to get IF and indexation in international databases. (PAME) on its behalf has taken an initiative in collaboration with University of Health Sciences (UHS) Lahore to start Certificate Course in Medical Editing (CME) to build professional capacity of the editors . This has helped many editors whose journals have now been recognized by the Higher Education Commission as per the latest revised list released last month.^4^,^5^

While we would like to thank our valued members of the Editorial Board and Reviewers who have played a vital role in the enhancement of our Impact Factor and improving the quality of manuscripts accepted for publication, we are mindful of our weaknesses as well. We need to increase our presence on Social Media which at present is not satisfactory. Accelerating the peer review system shortening the time from submission to acceptance and publication is also being looked into. We also need understanding by the authors whose papers are not accepted for various reasons and hope situation will improve in the days to come. Repeated lockdowns due to Covid19 Pandemic has also adversely affected our working. However, we were lucky to publish a special issue on Medical Imaging in collaboration with Wuhan Institute of Technology during September 2021 thereby further improving the academic collaboration and cooperation between the healthcare professionals and medical institutions of Peoples Republic of China and Pakistan.^6^This we believe will go a long way in further cementing our brotherly relations with China which has always proved to be a sincere friend of Pakistan. Our efforts continue to maintain the high standard and further improve upon our current Impact Factor in the coming years.

Yet another important step that Pakistan Journal of Medical Sciences has taken during the Year 2021 is collaborating with PharmEvo Pharmaceuticals to establish PharmEvo Research Forum with the main objective of promoting research culture and the art of medical writing and scientific publishing.^7^ An important component of this initiative apart from organizing Workshops and Training Courses is approval of Research Grants particularly for the young researchers, fellows in training who often have good ideas but fail to realize their objectives due to financial constraints. An Advisory Board consisting of eminent medical personalities selected from all over Pakistan will help decide issues related to grants approval on merit. Each grant will be for up to Rs. three hundred thousand. Workshops on proposal for grant writing are also being planned in different medical institutions all over the country in the coming months.

We have also offered Internship facility to one Fellow who is getting training for the last few months. We offer special prize, scholarships in the name of our founder Chief Editor late Dr. Maqbool H. Jafary to junior editors every years and some of the brilliant candidates in the CME Course by UHS are also picked up for this award. At the certificate distribution ceremony of CME Batch-2 held at UHS Lahore on June 23, 2021, four junior editors were presented this prestigious prize/award. This is our humble effort to promote the cause of medical journalism in Pakistan.
